# The structure of latherin, a surfactant allergen protein from horse sweat and saliva

**DOI:** 10.1098/rsif.2013.0453

**Published:** 2013-08-06

**Authors:** Steven J. Vance, Rhona E. McDonald, Alan Cooper, Brian O. Smith, Malcolm W. Kennedy

**Affiliations:** 1 School of Chemistry, University of Glasgow, Glasgow G12 8QQ, UK; 2 Institute of Biodiversity, Animal Health and Comparative Medicine, University of Glasgow, Glasgow G12 8QQ, UK; 3 Institute of Molecular, Cell and Systems Biology, University of Glasgow, Glasgow G12 8QQ, UK

**Keywords:** latherin, horse, sweat, surfactant protein, PLUNC proteins

## Abstract

Latherin is a highly surface-active allergen protein found in the sweat and saliva of horses and other equids. Its surfactant activity is intrinsic to the protein in its native form, and is manifest without associated lipids or glycosylation. Latherin probably functions as a wetting agent in evaporative cooling in horses, but it may also assist in mastication of fibrous food as well as inhibition of microbial biofilms. It is a member of the PLUNC family of proteins abundant in the oral cavity and saliva of mammals, one of which has also been shown to be a surfactant and capable of disrupting microbial biofilms. How these proteins work as surfactants while remaining soluble and cell membrane-compatible is not known. Nor have their structures previously been reported. We have used protein nuclear magnetic resonance spectroscopy to determine the conformation and dynamics of latherin in aqueous solution. The protein is a monomer in solution with a slightly curved cylindrical structure exhibiting a ‘super-roll’ motif comprising a four-stranded anti-parallel β-sheet and two opposing α-helices which twist along the long axis of the cylinder. One end of the molecule has prominent, flexible loops that contain a number of apolar amino acid side chains. This, together with previous biophysical observations, leads us to a plausible mechanism for surfactant activity in which the molecule is first localized to the non-polar interface via these loops, and then unfolds and flattens to expose its hydrophobic interior to the air or non-polar surface. Intrinsically surface-active proteins are relatively rare in nature, and this is the first structure of such a protein from mammals to be reported. Both its conformation and proposed method of action are different from other, non-mammalian surfactant proteins investigated so far.

## 1. Introduction

Surfactants occur widely in nature, typically involving small molecules such as bile acids, or the glycolipids and phospholipids that, in complex with small proteins, comprise the pulmonary surfactants of mammalian lungs. Proteins themselves rarely exhibit intrinsic surfactant activity except when misfolded or denatured, as commonly seen in laboratory preparations, in food products or in some fire retardant foams. Protein-based surfactants are of interest because they can be more efficient on a molar basis than small molecule surfactants yet still be compatible with cell membranes [1–4]. Examples of surfactant proteins that exhibit strong surface activity in their native state and in the absence of associated lipids or glycosylation include the hydrophobins of fungi [5], a protein found in the foam nests of certain amphibians (ranaspumin-2, RSN-2; [6]), and the subject of this report, the latherin protein of horses [3].

Latherin was originally described in the 1980s as an intrinsically surface-active, non-glycosylated protein that is abundant in horse sweat, and is the likely cause of the frothing seen in vigorously exercising animals [7]. It is produced and stored in granules in the sweat glands of skin, and is also synthesized in the salivary glands [3]. Its function is thought to be to wet the surface of the waterproofed hair shafts of horses to allow rapid movement and spreading of perspired water over the surface of the pelt for evaporative cooling [3,7]. This idea is reinforced by the demonstration that latherin will coat hydrophobic surfaces [3]. Horses, humans and patas monkeys (*Erythrocebus patas*) are the only mammals known to sweat copiously for thermoregulation, though the compositions of their sweat fluids differ significantly—humans have high salt, low protein sweat, whereas horses have high protein, low salt sweat [8–11]; the composition of patas monkey sweat has not been reported although their eccrine sweat glands are physiologically and morphologically similar to those of humans [11]. For equids, the combined surfactant and surface coating activities of latherin may be a special adaptation associated with their role as large, endurance-running flight animals that have a particular need to shed heat [8,9], a process otherwise impeded by a dense, hairy pelt. Latherin's presence in equine saliva, where it may also cause foaming, is more puzzling, but its wetting properties could assist mastication of, and salivary enzyme penetration into, the dry, coarse, fibrous diet for which equids are specialized. Also, its surfactant activity might directly control the establishment and growth of microbial biofilms on tooth or mucosal surfaces [12].

Latherin is the target of IgE antibodies in some, but not all people who are allergic to horses [3], and its primary structure contains peptide sequences of two previously classified horse dander allergens (Equ c 4 and Equ c 5; [13]), which were presumably cleavage fragments of latherin. A latherin-like allergen protein has also been characterized from the tongue epithelium and salivary glands of cats (Fel d 8; [14]). Whether latherin and its relatives are intrinsically allergenic, or become targets of allergic responses in certain individuals responding coordinately to other allergenic stimuli, remains to be seen. Solving the structure of a family of allergens for which no previous structural information is available will potentially contribute to the continuing search for a relationship between allergenicity and protein structures, despite the seeming unreliability of such predictions [15,16].

Latherin is unusually rich in non-polar amino acids (predominantly leucines), and its amino acid sequence allies it to the PLUNCs (palate, lung, nasal epithelium clones), a large and enigmatic family of proteins of unknown structure present in the oral, nasal and upper respiratory tracts of mammals [17]. The biological function of these proteins is poorly understood, though they have been postulated to be involved in innate immunity at mucosal surfaces [18]. None of the PLUNCs have been shown to have bacteriolytic or bacteriostatic activities, but one from humans (short (S)PLUNC1; new systematic name BPIFA1) exhibits a leucine content of similar order to latherin [3,19], is similarly surface-active, and has anti-microbial biofilm activity [12]. PLUNCs have also been implicated in defence responses to mycoplasma infection, allergic inflammation, as well as in homeostasis of the upper airway and in protection of the middle ear, although their mechanisms of action remain to be defined [20–22]. This postulated connection between PLUNCs and innate immunity is stimulated by their amino acid sequence similarities to larger, two-domain proteins that are directly involved in anti-bacterial activities: lipopolysaccharide-binding protein and bactericidal/permeability-increasing (BPI) protein [23]. These two proteins are similar to cholesteryl ester transfer protein (CETP) and phospholipid transfer protein [24–26], so there is a precedent within the larger protein family for interaction with hydrophobic entities, but not necessarily involvement in immune defence.

Understanding how intrinsically active surfactant proteins work requires a multidisciplinary approach, an essential part of which must be the determination of their macromolecular structures in bulk solution and how they may change at the interface with air or other surfaces with which they associate. We report here the structure of horse latherin, as determined by high-resolution nuclear magnetic resonance spectroscopy (NMR) in solution, and postulate how the structure may explain its surfactant activity. The structure of latherin differs significantly from those observed in other surfactant protein systems (the hydrophobins and ranaspumins). Latherin's mechanism of surfactant action might be similar to the ranaspumins and at least one member of the PLUNC family, but dissimilar to that of the hydrophobins.

## 2. Material and methods

### 2.1. Protein preparation

Recombinant protein was expressed from a synthetic latherin gene where codon usage was optimized for expression in *Escherichia coli* (GeneArt, Invitrogen). The gene was incorporated into expression vector pET-32 (Novagen) to produce protein with enterokinase-cleavable, N-terminal His_6_ and thioredoxin fusion tags. Expression was carried out in *E. coli* strain Tuner(DE3) (Novagen). Latherin was isolated from the soluble cell lysate by Ni-affinity chromatography, enterokinase cleavage, subtractive Ni-affinity chromatography and size-exclusion chromatography to yield pure protein (>95% by SDS-PAGE). Isotopically enriched latherin (^15^N, ^13^C) was prepared using M9 minimal medium incorporating ^15^NH_4_Cl and ^13^C_6_-d-glucose as the sole nitrogen and carbon sources. For collection of residual dipolar couplings (RDCs), a ^15^N-latherin sample was partially aligned by addition of filamentous phage Pf1 (Profos AG, Regensberg, Germany) at a final phage concentration of 5.0 mg ml^−1^ (10 Hz ^2^H splitting).

### 2.2. NMR data collection and assignment of spectra

NMR resonance assignment of ^15^N, ^13^C labelled latherin is described in detail elsewhere [27]. All spectra were recorded at 310 K in 20 mM sodium phosphate, 50 mM NaCl, 1 mM NaN_3_, pH 7.5 on a 14.1 T Bruker AVANCE spectrometer equipped with a Cryoprobe. Standard triple resonance experiments were supplemented with methyl-specific TOCSY experiments [28] to aid assignment of the high number of leucine residues. Spectra were processed using Azara (Wayne Boucher, Department of Biochemistry, University of Cambridge, http://www.bio.cam.ac.uk/azara) and analysed using CcpNmr Analysis v. 2 [29].

### 2.3. Structure calculation

Nuclear Overhauser effect (NOE) restraints were created from three-dimensional ^15^N-NOESY-HSQC and ^13^C-edited ^1^H,^1^H spectra each with 100 ms mixing time. Distance restraints were derived from NOESY crosspeaks with the initial mapping from normalized intensity to distance following a 1/r^6^ relationship. NOE distance restraints were incorporated in restrained molecular dynamics calculations using the ambiguous distance restraints formalism [30]. Estimates of the average contribution of the dipolar coupling to J_NH_ (and the associated error) were obtained by collecting two independent IPAP-[^15^N]-HSQC datasets from both isotropic and anisotropic samples. The magnitudes of the axial and rhombic components of the alignment tensor were estimated using the method described by Clore *et al*. [31]. Eighty-eight D_NH_ restraints were incorporated into the structure calculations via the SANI potential [32] in square-well mode. Thirty hydrogen bond restraints were included for amide protons where signals were still observed in a ^15^N-HSQC recorded 20 min after dilution of a ^15^N-latherin sample into 90% (v/v) D_2_O. Hydrogen-bond acceptors were identified by inspection of the NOE-refined structures, supported by NOE data. Restraints for the conserved disulfide bond were introduced once juxtaposition of the cysteine residues was observed in structure calculations.

Structures were calculated from randomized initial atomic coordinates using CNS [33] with the PARALLHDG-5.3 force field with PROLSQ non-bonded energy terms [34]. Initial structures were subsequently refined by iteratively filtering the ambiguous distance restraints against the calculated structures to discard duplicate restraints and assignments contributing less than one to five per cent to the total NOE intensity. RDC and hydrogen bond restraints were then introduced. *ϕ*, *φ* dihedral angle restraints for areas of regular secondary structure, produced using DANGLE (Cheung, University of Cambridge, http://dangle.sourceforge.net/; [35]) were included within initial stages of structure calculation to aid convergence and then omitted from final cooling steps. After eight rounds of NOE disambiguation using ARIA v. 2.3 [36], the 20 lowest energy models from a final round of 100 calculated structures were refined in explicit water. These 20 models were then used to create the representative ensemble of structures. The quality of these structures was analysed using PROCHECK [37] and their coordinates deposited in the Protein Data Bank (www.wwpdb.org) under accession code 3ZPM.

### 2.4. ^15^N Relaxation measurements

N-relaxation rates, R1 and R2 were assessed using the method of Kay and co-workers [38–40] at a field strength of 600 MHz. Relaxation delays for assessment of R1 were 1200, 1600, 2100 and 2600 ms while those for R2 were 17, 34, 68, 102 and 136 ms. The first and third experiments in each series were repeated in order to estimate the inherent error in calculation of crosspeak intensities. Relaxation times T1 and T2 were calculated using nonlinear least-squares fitting. Collection of ^15^N-HSQC-heteronuclear NOE experiments with and without saturation allowed extraction of [^1^H]^15^N NOE values. Both saturation and reference experiments were repeated for the purpose of error estimation. The rotational correlation time, τ_m_, for each amide residue was calculated using the method described by Kay *et al*. [39]. The rotational diffusion tensor of the latherin molecule was then calculated via the quadric representation approach proposed by Bruschweiler *et al*. [41] and Lee *et al*. [42] using the *quadric_diffusion* program (Palmer III, www.palmer.hs.columbia.edu/software.html). The model-free formalism as described by Lipari & Szabo [43,44] was used to determine the amplitudes and timescales of intramolecular motions of the latherin backbone from the three relaxation parameters. This analysis was carried out using the *FAST ModelFree* program (Loria, Yale University, http://xbeams.chem.yale.edu/~loria/software.php; [45]).

### 2.5. Hydrogen–deuterium exchange

Hydrogen–deuterium exchange rates were calculated by rapid dilution of a ^15^N-latherin sample to 90% (v/v) D_2_O. ^15^N-HSQC spectra were collected at 20 min intervals for 3 h and then at 1 h intervals for a further 5 h (see examples in the electronic supplementary material, figure S1). Residues that displayed exchange on the timescale of the experiment were assigned as undergoing medium exchange. Residues that had reduced to 10 per cent of the original intensity (relative to a reference) within the 20 min required to record the first spectrum were assigned as undergoing fast exchange. Residues that displayed no change of intensity (relative to a reference) within the 8 h were assigned as undergoing slow exchange. Hydrogen–deuterium exchange rates (*R*_H−D_) for medium exchanging residues were calculated by fitting peak intensity (*I*) against time (*t*) with *I*_t_/*I*_0_ = exp(−*R*_H−D_.*t*) + *c*.

## 3. Results

### 3.1. Latherin has a BPIF ‘super-roll’ structure in solution

**Figure 1.**
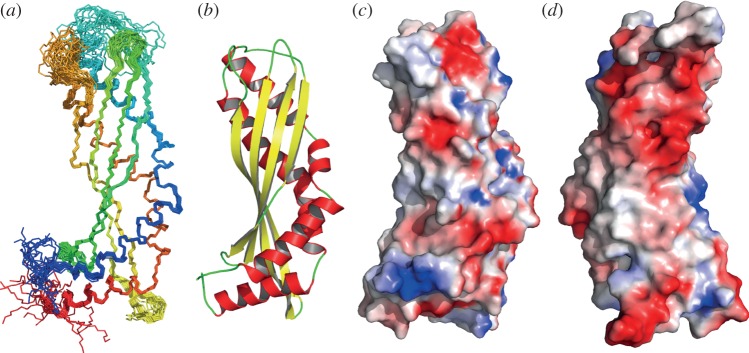
The solution structure of latherin. (*a*) The ensemble of the 20 latherin models (superimposed) that best fit the experimental data, shown in peptide backbone representation, shaded from blue (N-terminus) to red (C-terminus). (*b*) Ribbon model of the representative structure of latherin in solution illustrating secondary structure elements; α-helices are coloured red and β-strands yellow. (*c*) Surface contact potential (blue, positive; red, negative) of latherin mapped on the solvent accessible surface of the protein in the same orientation as (*a*), and (*d*) rotated 180°. Images and contact potential generated using PyMOL [46].

Purified recombinant latherin, which has previously been found to exhibit properties in solution similar to the natural material [3,7], exhibited sufficiently sharp, well-resolved NMR spectra suitable for high-resolution structure determination after appropriate isotopic (^13^C, ^15^N) enrichment. Potential difficulties due to spectral overlap arising from the high proportion of leucine residues were overcome as described in §2 and [27]. A total of 6922 NOE-derived distance restraints were used to calculate the structure of latherin, of which 4293 were unambiguous or manually assigned, with 2210 ambiguous restraints in the final refinement (table 1). These were supplemented by 88 RDC restraints and 34 hydrogen bond restraints. The structure calculations converged well to give good agreement with the experimental data and a tightly defined ensemble of structures (see the electronic supplementary material, tables S1 and S2; figure 1*a*).

**Table 1.** Experimental restraints and statistics of the calculated structures. Average statistics were calculated from the 20 water-refined structures in the latherin ensemble. The number of violations is shown as the average and standard deviation per structure.

NOE distance restraints	
NOE restraints	6503
ambiguous	2210
unambiguous	4293
intraresidue	1985
interresidue	2308
sequential (*i* − *j* = 1)	985
medium-range (*i* − *j* < 5)	518
long-range (*i* − *j* > 5)	805
violations per structure >0.5 Å	1.15
violations per structure >0.3 Å	8.60
distance restraint RMSD	0.036 Å
other restraints	
RDCs	88
RDC Q factor	0.127
hydrogen bonds	34
dihedral angle restraints	369
disulfide bond	1

Latherin is monomeric in solution and exhibits an almost cylindrical structure about 65 Å long by 25 Å wide (figure 1*a*,*b*). These dimensions are in good agreement with the Stokes radius estimates for natural latherin yielding an axial ratio of approximately 3 : 1 [7]. Latherin comprises a four-stranded β-sheet against which two long anti-parallel helical regions pack, following the groove in the concave face of the β-sheet. The N- and C-termini are found at one end of the cylinder (the ‘terminal end’), where a third short helical region (helix α_C_) is angled across the diameter of the molecule. The other end of the molecule (the ‘loop end’) is distinguished by three extensive flexible loops that are notable in their relatively high content of exposed apolar side chains, in particular leucines.

**Figure 2.**
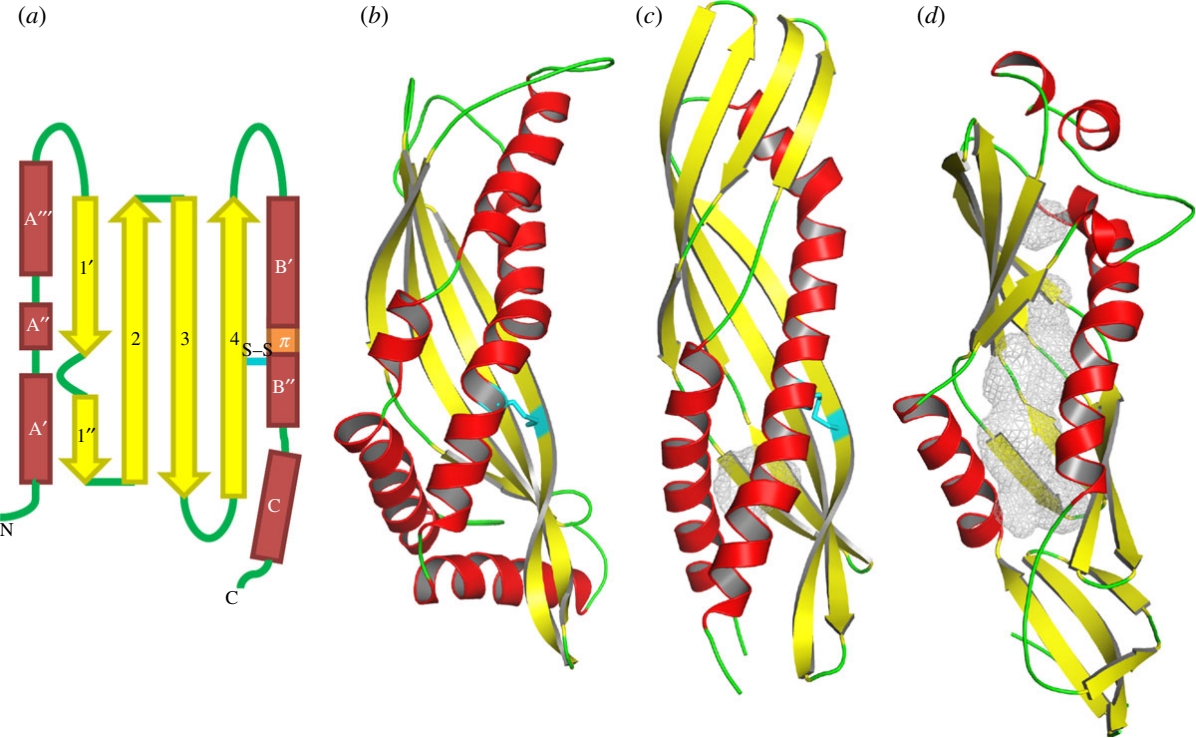
Topology of latherin and comparison with BPI. (*a*) Topology model of latherin. α-Helices are represented by red rectangles; β-strands by yellow arrows; non-regular secondary structure as green lines. The intramolecular disulfide bond, Cys133–Cys175, is shown as a cyan line labelled ‘S–S’. The short section of π-helix is coloured orange, and the β-bulge by a curved green line between strands 1′ and 1″. (*b*) Cartoon representation of latherin compared with, (*c*) and (*d*), the N- and C-terminal domains, respectively, of BPI protein (PDB code 1BP1; [47]). The grey mesh in (*d*) encloses the internal cavities in the BPI C-terminal domain that are accessible to a 1.4 Å radius probe. Images created using PyMOL [46].

The N-terminal helix, labelled α_A_, stretches from residues 7–47 with two breaks in the regular secondary structure at residues 22–23 and 31–33. Helix α_A_ can therefore be subdivided into three sections: 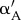
(7–21), 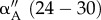 and 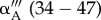 (see topology diagram in figure 2*a*). Helix α_B_ (152–203) is also interrupted such that 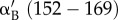 and 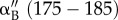 are separated by a short region of π-helix as indicated by the signature *i* to *i* + 5 hydrogen bonding between the amides of residues 171–172 and the carbonyl groups of 166–167, respectively. Sections of π-helix can destabilize a helix and are often associated with functional sites [48–51]. Helix α_C_ comprises residues 188–203. The four strands that make up the anti-parallel β-sheet are β_1_ (61–77), β_2_ (83–97), β_3_ (104–120) and β_4_ (126–142). β_1_ is interrupted by a β-bulge at a point where a proline (Pro89) in strand 2 introduces an irregularity in the packing of the two strands and does not present a hydrogen bonding partner to strand β1. It can therefore be divided into two sections 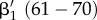 and 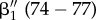. The disulfide bond (Cys133–Cys176) connects β_4_ to 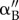.

The interior of the protein is notable for its paucity of polar side chains. The single exception is Thr110, which is closely surrounded by apolar aliphatic amino acids. Its position there, however, may be stabilized by hydrogen bonding between its hydroxyl to the main chain carbonyl of Leu136.

As expected from amino acid sequence comparisons [17,23], database examinations identify the fold adopted by latherin as a BPI domain-like fold (SCOP; [52], code 55393) or as a super-roll (CATH; [53], code 3.15). A search for other proteins with related folds using DALI [54] identified several structures with better than marginal match scores (*z* score >5). In addition to the structures of BPI and CETP, the search identified several similar structures from outwith the cognate BPI superfamily of proteins, including Der p 7, a dust mite allergen [55], juvenile hormone-binding protein (JHBP; [56]) and takeout protein 1 (Top1; [57]), all of which are involved in binding hydrophobic ligands, Aha1, which is apparently functionally unrelated, being an intracellular co-chaperone of the molecular chaperone, Hsp90 [58], and Yceb, which is an as yet uncharacterized lipoprotein from *E. coli* (protein structure database (PDB) code 3L6I). β-strands in latherin are shorter than those of the other proteins with a similar fold, with the result that the β-sheet at the ‘loop end’ does not twist as far around the helices. The single disulfide bond, which links the final strand of the β-sheet to the C-terminal helix in latherin, is a feature found in an analogous position in the N-terminal domains of BPI and CETP, as well as JHBP. Latherin is unique among the members of the superfamily in having two helical regions juxtaposed for the entire length of the concave face of the β-sheet.

### 3.2. Latherin is not obviously amphiphilic but has surface exposed hydrophobic residues at one end

**Figure 3.**
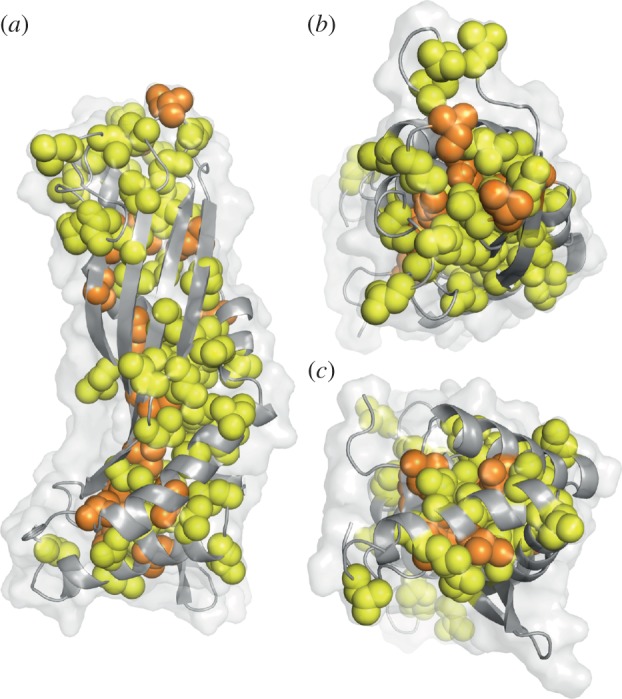
Distribution of leucine and isoleucine residues in the latherin structure. Latherin's main chain is displayed in cartoon representation, with the solvent accessible surface envelope shown in transparency. Leucine side chains are displayed as yellow spheres, and isoleucine side chains as orange spheres. In (*a*), the ‘loop’ end is at the top and the ‘termini’ end at the bottom. (*b*) and (*c*) show views from the ‘loop’ and ‘termini’ ends, respectively. Leucine and isoleucine residues predominantly line the core of the fold except at the ‘loop’ end. Image created using PyMOL [46].

The structure of latherin displays little evidence of any amphiphilicity that might have been anticipated by comparison with the distinct patches of polar and apolar side chains seen on the surface of hydrophobins [59–61]. By contrast, the exterior of latherin in the bulk phase, shows no such surface patches, being almost exclusively decorated with the side chains of hydrophilic residues and predominantly anionic due to the higher proportion of aspartic and glutamic acids over arginines, histidines and lysines (figure 1*c*,*d*). This is intriguing and initially unexpected, especially in view of the unusually high proportion of leucines in latherin (49 of the 208 residues), a trait common to human SPLUNC1, which also exhibits surfactant activity [12]. In the latherin structure, the leucines are evenly distributed along the length of the structure, being mainly confined to the interior in the ordered regions of the protein (figure 3*a*). But, at the loop end, about one-third of all the leucines are exposed to solvent (cf. figure 3*b* for loop end, and figure 3*c* for termini end; and see the electronic supplementary material, table S3 for numerical comparison). That these loop leucines and other adjacent aliphatic residues do not form an obvious hydrophobic patch is in part because their polar main-chain groups are solvent exposed and also because they are interspersed with polar residues.

We previously detected no interaction between latherin and 8-anilino-1-naphthalenesulfonic acid (ANS), a small fluorescent probe for exposed apolar regions or pockets in a protein [3], which might otherwise be expected of an amphiphilic molecule. Analysis of the latherin structure, however, revealed no evidence for cavities or pockets to which ANS might bind when in bulk solution.

### 3.3. Latherin is well ordered on the picosecond to nanosecond timescale with a few dynamic loops

The lack of obvious surface-exposed hydrophobic regions on latherin, its monomeric state in solution and our previous neutron reflection findings [3], suggest that a radical conformational change is required for latherin to facilitate surface tension reduction and association at an air : water or non-polar interface. This is likely to be reflected in features of the protein in the bulk phase observable as regions exhibiting unusual dynamic properties. The molecular dynamics of latherin in solution was therefore investigated using two methods: firstly, by examining the relaxation dynamics of its backbone amides for evidence of regions with high internal motion; secondly, by monitoring the rates of solvent exchange of labile hydrogens when dissolved in D_2_O.

**Figure 4.**
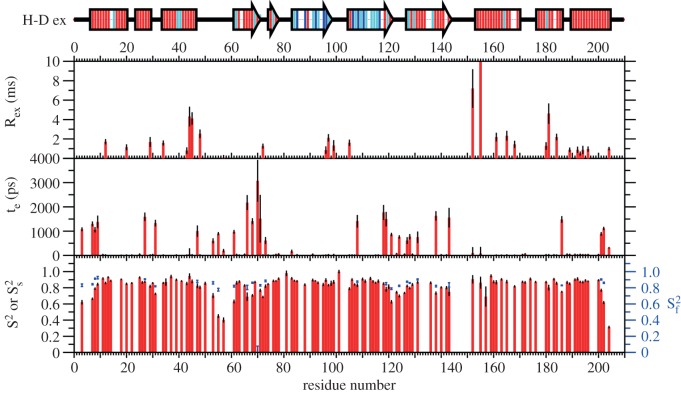
Latherin backbone dynamics. The Lipari–Szabo extended model-free parameters derived from ^15^N relaxation measurements at 14.1 T are plotted by residue number in the three graphs. Backbone amide hydrogen–deuterium exchange rates indicated on the secondary structure schematic above, with residues undergoing fast exchange (lifetime <20 min) coloured red, medium exchange (20–480 minutes) cyan and slow exchange (>480 min) blue in the secondary structure cartoon.

Latherin's backbone dynamics were analysed using the Lipari–Szabo model free approach based on amide ^15^N relaxation measurements. The data were best fit with an overall correlation time of 11.3 ns and an axially symmetric diffusion tensor with a *D*_||_/*D*_⊥_ ratio of 1.68 to obtain order parameters, local correlation times and exchange broadening terms (figure 4), revealing that the four β-strands show low levels of internal motion, with the exception of the residues preceding (Gln66, Thr68 and Leu70) and within the β bulge (Leu71, Gln72 and Leu73), and those preceding and following the short loop between β_3_ and β_4_ at the terminal end. The two termini are themselves dynamic, as is the nearby loop (121–125), though the other loop in this region (residues 78–82) shows relatively little internal motion. At the other end of the molecule, the loop between α4 and β1 (48–60) exhibits depressed order parameters indicating a high degree of flexibility that correlates with the poor definition of this part of the structure. The shorter loop between β2 and β3 (98–103), in contrast, displays motion on the millisecond timescale for the residues for which relaxation data can be obtained. The long loop between β_4_ and 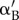 (143–151) could not be examined directly because of the absence of amide crosspeaks for residues in this region (although Gln143 at one end of the loop was seen to be dynamic), which may itself be evidence of a substantial degree of motion.

Hydrogen–deuterium change experiments also revealed significant differences between various parts of the molecule. Notably, all of the residues showing slow rates of exchange, and most of those showing medium rates, occur in the two central strands β2 and β3, consistent with the relaxation dynamics data. The remainder of the molecule, including the three main helical regions, undergoes fast exchange, with only a few isolated regions at the centre of each α-helical segment exhibiting lower rates of exchange. The hydrogen–deuterium exchange results therefore agree well with the relaxation dynamics analysis, further demonstrating the stability of the two central strands of the β-sheet.

In summary, latherin is a molecule with a more rigid core and two dynamic ends, the loop end in particular having three loops which show high levels of chemical exchange.

## 4. Discussion

### 4.1. Latherin's structure and possible conformation change at an interface

The three-dimensional molecular structure of latherin in its aqueous solution phase described here is the first such structure reported for an intrinsically surfactant protein from mammals. This new information promises a broader understanding of how proteins can exhibit strong surfactant properties in their native states without the involvement of other cofactors such as lipids or glycans. As we and others have shown, naturally occurring surfactant proteins such as hydrophobins, ranaspumins and latherin exhibit significant surfactant properties at concentrations several orders of magnitude lower than usually observed with other proteins. Moreover, as reviewed in [4], this surfactant activity is directly related to structure, either because of amphiphilicity (as in the case of hydrophobins) or clam-shell/hinge-opening (as postulated for RSN-2), where the native conformation predisposes such proteins to biologically significant surface interaction. This is clearly distinct from that which is observed in the non-specific interfacial unfolding of other proteins, which usually takes much longer to appear and often requires quite aggressive denaturation treatment depending on the protein. The overall structure and molecular surface properties of the latherin molecule are radically different from those of other surfactant proteins known to date, and suggest yet another means of achieving spontaneous protein surfactant activity.

**Figure 5.**
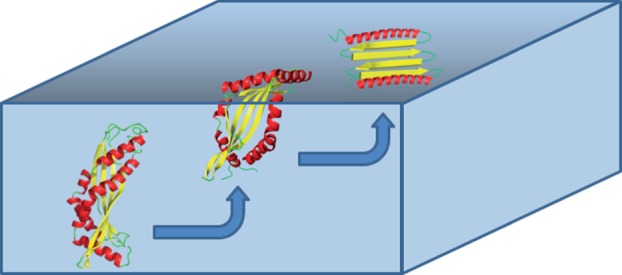
Latherin unfolding at an air : water interface. Speculative model of how latherin may transform from its fold in the bulk phase to an opened-out conformation at an air : water interface, thereby exposing its apolar interior to the air. The model shows three stages, from left to right: latherin in the bulk phase in which recognition of the interface occurs via the relatively hydrophobic loops; initial unzipping of the two α-helices initiated from the ‘loop’ end and a final open, planar, conformation, retaining secondary structure but with the hydrophobic core exposed at the interface. There will likely be dynamic exchange between the three conformations. A similar process may apply for latherin associating with a hydrophobic solid surface.

As we have observed elsewhere [6], the structure of a surfactant protein in bulk solution does not necessarily reflect its disposition at the air : water interface. Indeed, for monomeric proteins in solution, conformational change at the interface would seem to be a requirement in order to reconcile the need for good aqueous solubility in the bulk phase, while presenting a more amphipathic appearance at an interface. Such a radical conformational change also seems to be required for latherin. Our previous neutron reflection data indicate that latherin forms a relatively thin (mono)layer approximately 10 Å deep at the air : water surface, and with an area of approximately 4350 Å^2^ per molecule [3]. Interestingly, neutron reflection studies show that non-specific irreversible interfacial layers sometimes observed with other proteins are much thicker in comparison, typically of order 30 Å [62–65]. The latherin cylinder in the bulk phase is about 75 Å long by about 25 Å in diameter, which is incompatible with a 10 Å layer in its fold in the bulk phase. Complete unfolding and flattening of a cylinder of these dimensions yields an area of approximately 5890 Å^2^, which, while accepting the crudity of this approximation, is compatible with the value obtained from neutron reflection. How latherin initially associates with an interface, and the events that follow, remain unknown, but its structure and dynamics provide both clues and topological constraints. The dynamic, unstructured, apolar side chain-rich loops are the most likely place where the protein could associate, penetrate and anchor to a surface, and the loops would have sufficient flexibility to then splay out with apolar side chains oriented towards the air or a non-polar solid substrate. Subsequent unzippering of the protein cylinder is unlikely to occur between any of the β strands because of the cooperative hydrogen bonding between them. This constraint is reinforced by the hydrogen–deuterium exchange data that identifies the inter-strand H-bonds (in particular, between strands 2 and 3) as the most stable in the molecule. Unstitching between strand 4 and helix B is unlikely given the disulfide bond that connects them approximately midway down their lengths. Assuming minimal change in the secondary structure elements, this leaves the seams between the two helices, or between helix A and strand 1 as likely fault lines. Given that the solvent-excluded interfacial area buried between the two helices is approximately 2000 Å^2^, and that between helix A and strand 1 is approximately 1300 Å^2^, the latter case appears to be the more favourable. Conversely, assuming that unfolding initiates from the apolar loops, as proposed above, the ability of helix A and strand 1 to reorient in an independent manner is likely to be inhibited by the loop that connects the two features at this end of the protein. By contrast, there is no such topological constraint on the relative orientation of the two helices. A possible unfolding sequence involving an opening between the helices is illustrated in figure 5.

### 4.2. Latherin operates as a surfactant differently from hydrophobins

The mechanism by which latherin operates as a surfactant is clearly different to that of the hydrophobins. These are rigid, amphiphilic molecules with a distinct surface patch of apolar amino acid side chains on the face of each molecule [5,60,61]. In solution, they form oligomers in which the hydrophobic patches are isolated from solvent water, allowing the proteins to remain in solution as dimers or tetramers [5]. At an air : water interface, hydrophobins orientate with the apolar surfaces projecting into air, whereas the polar regions remain immersed in the water phase. Charge interactions on the flanks of adjacent hydrophobin molecules yields self-associating monolayers [5] without the necessity for any conformational change. Latherin, in contrast, remains monomeric in solution, and appears more akin to the frog foam nest protein, RSN-2 in this respect (though with a quite different fold) and also appears to undergo significant conformational change at an air : water interface [6].

### 4.3. Unusual environment of latherin's single tryptophan side chain

**Figure 6.**
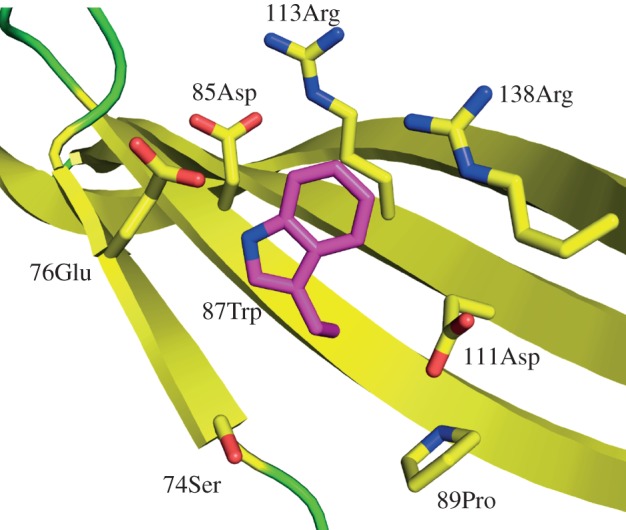
The environment of the solvent exposed tryptophan. Trp87 and surrounding side chains are shown in stick representation, with side-chain oxygens and nitrogens coloured red and blue, respectively. Image created using PyMOL [46].

One puzzle presented by our previous work on latherin relates to the fluorescence properties of its single tryptophan residue (Trp87 in the structure; [3]). Latherin in dilute aqueous solution exhibits a relatively red-shifted Trp fluorescence emission spectrum, usually indicative of exposure to solvent water or a charged local protein environment [66,67]. Quenching of fluorescence emission by Trp87 by neutrally charged compounds (succinimide, acrylamide) was efficient, consistent with side-chain exposure to polar solvent water [3]. But, quenching by iodide (I^−^), normally a highly efficient quenching agent for exposed Trp residues, was unexpectedly ineffective [3]. The new structure explains this conundrum in that Trp87 is exposed on the exterior of the protein, midway down the concave side of the curved cylinder, with its indole side chain sandwiched between, and encircled by, nearby charged amino acid side chains (Asp85, Asp111, Arg113, Arg138 and Glu76; figure 6). So, Trp87 is in a position to encounter solvent water and be quenched by neutral compounds, but its local environment is sufficiently dominated by negatively charged groups to repel a normally highly efficient but negatively charged quencher.

### 4.4. Structural similarities and differences between latherin and its relatives

Although relatively uncommon, the latherin super-roll fold has been observed elsewhere, most notably as part of much larger proteins such as in domains of the BPI and CETPs of humans [24,68]. Both of these proteins have two latherin-like domains, the N-terminal domain of each being similar to latherin, with the C-terminal domains appearing to have diverged in structure (see figure 2 and electronic supplementary material, S2). This similarity is nicely illustrated in a superposition of latherin and BPI's C-terminal domain (not shown). Interestingly, the short section of π-helix in the 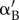 helical region of latherin is a feature shared in the C-terminal helices of both domains of BPI and CETP, and π-helices have been proposed to have some role in conformational exchange associated with function in CETP [51].

In contrast to the ligand-binding cavities seen in BPI and CETP, the close packing of large, apolar amino acid side chains in the hydrophobic core of latherin leaves no internal cavity. BPI is, like latherin, a long, slightly curved cylinder, of similar diameter to latherin but about twice as long. It has two latherin-like domains that are fused closely end-to-end, with some of their chains intertwining, forming a single piece, boomerang-shaped molecule. Each of the domains exhibits latherin-like folds, the N-terminal domain particularly so. These proteins interact with lipids, and CETP is known to have a cavity into or through which lipids may move [51]. So, it is conceivable that both BPI and CETP arose from domain duplications of latherin- and PLUNC-like ancestors, followed by specialization and structural alteration of one (BPI) or both (CETP) domains. Their presumptive descent from a common ancestor protein is also indicated by similarities in the intron positions in their encoding genes [17].

Perhaps, the most interesting comparison would be between the structure of latherin and the PLUNCs, which are, like latherin, single domain members of the BPI superfamily. One of these from humans (BPIFA1; SwissProt Q9NP55) is highly expressed in the trachea, progressively less so from proximal (bronchial) to distal (bronchiolar) airways, and, as latherin, has an unusually high content of leucines and exhibits surfactant activity [12,19]. No structure for a PLUNC is yet available, so we attempted here to model BPIFA1 using latherin as a template. Despite the familial affiliation, a simple alignment of the two sequences illustrates how divergent the two are, and latherin exhibits several amino acid position deletions relative to human BPIFA1 (see the electronic supplementary material, figure S3). Nevertheless, a reasonably acceptable model could be created which is similar in overall structure to that predicted previously using the X-ray crystal structure of the much larger BPI (PDB, accession 1BP1) as template [17]. The current model suggests that this PLUNC may share with latherin the long unstructured loop regions at the end of the molecule equivalent to latherin's loop end, together with a similar distribution and concentration of leucine residues (see the electronic supplementary material, figure S4A). With regards to the mechanism of its surface activity, the model of BPIFA1, as with our empirical structure for latherin, shows no sign of patches of hydrophobic or charged amino acid side chains exposed on its surface (see the electronic supplementary material, figure S4B). The corresponding regions of several members of the PLUNC/BPI family have been identified as key motifs in their ability to bind the target lipid [26,69,70]. The proposition that these loops are involved in surface detection in latherin may infer a similar mode of substrate recognition throughout the BPIF superfamily, and we cannot at this stage rule out the possibility that latherin may interact with lipids.

### 4.5. Other ‘super-roll’ proteins: common ancestry or convergent evolution?

A wider search for non-mammalian proteins with a similar fold to latherin yielded several structures. These derive from a wide range of eukaryotic taxa, and a pertinent question would be whether they represent true descent from an ancient common ancestor protein, or cases of convergent evolution. These proteins are found in insects (a juvenile hormone-binding protein, and a lipid-binding takeout protein in lepidopterans [57]); arachnids (the Der p 7 allergen of a house dust mite [55]) and in an apicomplexan, the malaria parasite *Plasmodium falciparum* (AHA-1; activator of Hsp90 ATPase from yeast; [58]; see the electronic supplementary material, figure S2 for comparisons). These show distinct structural similarities to the BPI/CETP/PLUNC/latherin (BPIFA) family of mammals, despite highly divergent amino acid sequences, so the question arises as to whether they represent true descendents of an ancestral protein, or a case of convergent evolution. The argument for descent from a common ancestor is most convincing for the mammalian and insect proteins, whose structures most closely resemble that of latherin in that they have intramolecular disulfide bonds in similar positions, and their encoding genes have similar arrangements of introns [17,23]. Der p 7 and AHA-1 resemble latherin the least, so may represent independent evolution of a latherin-like super-roll fold, but there is as yet insufficient phylogenetic and protein bioinformatic information of these proteins in such diverse taxa to be sure that missing links do not exist.

## 5. Conclusions

The structure of latherin confirms that it is a member of a family of proteins in mammals with similarities in their structures, but a remarkable divergence in their biological functions. Its closest relatives are synthesized in the salivary glands, oral cavity and associated structures, so latherin possibly evolved from a PLUNC-like ancestor as a specialization in equids for processing dry, fibrous dietary materials, and/or to control microbial biofilms on teeth and mucosal surfaces. While continuing to perform such functions, it may then have been recruited to the skin as equids evolved into large-bodied flight animals capable of sustained exercise requiring rapid onset and efficient heat dissipation. Latherin represents the first intrinsically surfactant protein of mammals whose structure is known, but, more, it reveals a potential mechanism of action that has not been demonstrated before for an animal protein in its native state, with the exception of the RSN-2 frog foam nest protein [6], but clearly different from that of other classes of surfactant proteins whose structures are known [5,61]. Consequently, this is of general interest across a broad range of disciplines including not only protein structural biology and biophysics, but also having potential implications in veterinary science, human health and bio- and nano-technologies involving protein–surface interactions.
